# An Introductory Curriculum for Internal Medicine Interns in Point-of-Care Ultrasound to Detect Lower Extremity Deep Vein Thrombosis

**DOI:** 10.24908/pocus.v7i2.15937

**Published:** 2022-11-21

**Authors:** Peter C Nauka, Darlene LeFrancois, Benjamin T Galen

**Affiliations:** 1 Division of Pulmonary, Allergy and Critical Care Medicine, Department of Medicine, University of Pittsburgh Medical Center Pennsylvania, Pittsburgh USA; 2 Department of Internal Medicine, Division of General Internal Medicine, Albert Einstein College of Medicine and Montefiore Medical Center Bronx, NY USA; 3 Department of Internal Medicine, Division of Hospital Medicine, Albert Einstein College of Medicine and Montefiore Medical Center Bronx, NY USA

**Keywords:** POCUS, DVT

## Innovations in POCUS Curriculum

Lower extremity deep venous thrombosis (DVT) is prevalent amongst hospitalized patients and is associated with significant morbidity and mortality 1. Delays in DVT diagnosis may result from relying on duplex ultrasound studies performed and interpreted by the vascular laboratory 2. The point-of-care ultrasound (POCUS) evaluation for DVT, specifically two- and three-point compression ultrasonography, offers excellent predictive value for proximal, above-knee DVT with sensitivity and specificity consistently >90-95% across many studies 3. 

POCUS for DVT has been utilized by non-radiologist physicians and non-vascular technicians and validated in various settings including the emergency department, intensive care unit and hospital floor 4, 5, 6. There is significant interest in incorporating specific training for internal medicine residents. Major barriers to implementing POCUS education within internal medicine residency curriculum include time constraints in busy didactic schedules, need for multiple trained facilitators, learners’ uneven prior experience with ultrasound, and access to machines. In this brief report, we highlight a standardized one-hour POCUS for DVT curriculum that can be administered by one or two facilitators. This format can be easily incorporated into most intern didactic schedules, e.g. “afternoon report.” 

Our curriculum consisted of a half-hour didactic session followed by a half-hour session of facilitated scanning on inpatients who were identified prior to the session. The didactic session included a review of ultrasound basics as well as a detailed overview of the POCUS for DVT protocol including video clips of negative and positive scans (Supplementary File S1). Beyond describing the protocol, specific attention was focused on proper identification of vascular anatomy, potential DVT mimics, as well as limitations of POCUS for DVT. 

The second half of the session focused on facilitated scanning of inpatients who were identified and amenable to practice scans prior to the session. These patients had lower extremity duplex ultrasound exams ordered or already completed prior to the educational session to allow for post scanning correlation of POCUS findings. We found that that a facilitator could be paired with approximately five or six interns at the bedside during a half-hour scanning session, which allowed all learners a chance to practice compression ultrasonography. Appropriate facilitators included credentialed faculty or senior residents with prior experience with POCUS for DVT. Facilitators were provided with a validated checklist of procedural steps to observe and evaluate interns’ ability to correctly identify vascular anatomy (Supplementary File S2). While we did not utilize the checklist beyond serving as a tool to ensure immediate training efficacy regarding skill acquisition, it may serve as a useful instrument to validate this or similar curriculum and longitudinally determine if learners are retaining material appropriately. 

Time constraints within the resident didactic schedule are a remaining barrier to incorporating POCUS for DVT. This one-hour introductory curriculum provides a time and resource effective approach to teach the three-point compression protocol to residents. We believe that this model can be easily incorporated into didactic schedules of most residency programs. Further study is needed to determine whether this curriculum is effective for achieving competency in the POCUS exam for DVT detection and longitudinal skill retention. 

## Disclosures

None.

## Supplementary Material

Supplementary File S1

Supplementary File S2
