# Supplementary material for: An Introductory Curriculum for Internal Medicine Interns in Point-of-Care Ultrasound to Detect Lower Extremity Deep Vein Thrombosis
Source: POCUS J. 2022 Nov 21;7(2):185–6. doi: 10.24908/pocus.v7i2.15937 (PMC9983714; doi:10.24908/pocus.v7i2.15937)
Supplement: Supplementary File S1 [file pocusj-07-15937-s001.pdf]

DVT Detection by Point-of-Care Ultrasound (POCUS)

Peter Nauka, MD

Benjamin Galen, MD

EINSTEIN
Albert Einstein College of Medicine

Montefiore
THE UNIVERSITY HOSPITAL FOR ALBERT EINSTEIN COLLEGE OF MEDICINE

A few points about POCUS

What it is?

- Using ultrasound at bedside by clinician (you!)
- Gives more information to justify a differential diagnosis
- Extension of physical examination
- Helpful tool when diagnosis is unknown or unclear

What it is not?

- Not the gold standard for detecting DVT
- Should not supplant an official duplex
- Findings should not be used in isolation (ex. Would not assume a patient has a VTE solely on positive D-dimer)

Ultrasound Basics

- Probe emits and detects refracted ultrasound waves.

- What we see on screen depends on tissue density:
  - Blood vessels – hypoechoic (“dark” or “black”)
  - Muscle/Subcutaneous tissue – hyperechoic (“gray” or “bright”)

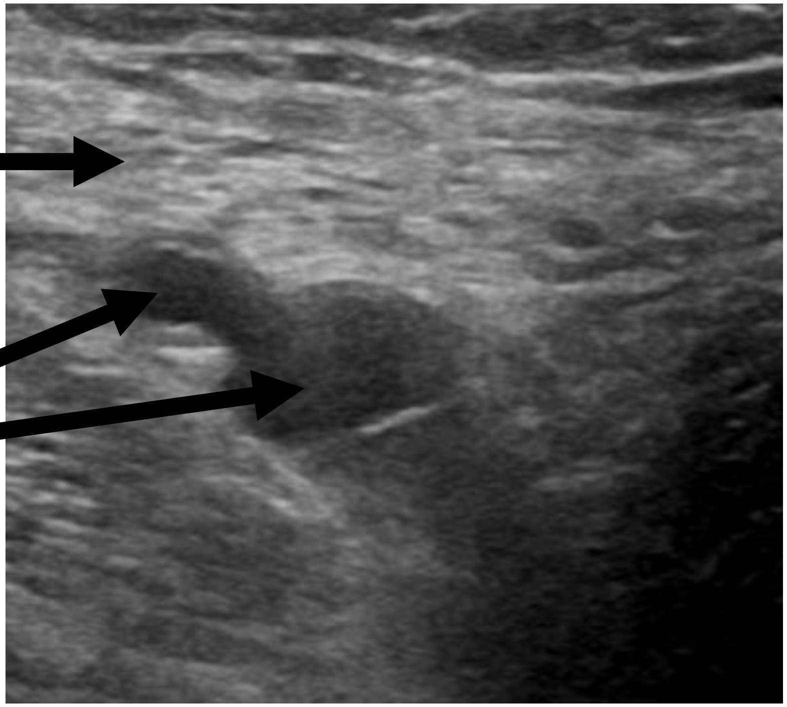

How do we differentiate between arteries and veins?

3-point compression Ultrasound

- There are many ways we can detect thrombi using ultrasound, but the simplest way is using **compression.**
  - Veins with thrombi will not be *fully* compressible

- We will focus on the 3-point compression ultrasound (3-CUS) protocol.

- 3-CUS has excellent sensitivity and specificity
  - Hospitalists -> HOCUS-POCUS (n=73): Sensitivity 100%, Specificity 95.8%
  - ED Physicians -> Garcia et. al (n=109): Sensitivity 93.2%, Specificity 90.0%
  - Critical Care Fellows -> Kory et. al (n=129): Sensitivity 86%, Specificity 96% (this was using only 2-point compression!)

Needleman L, Cronan JJ, Lilly MP, et al. Ultrasound for Lower Extremity Deep Venous Thrombosis: Multidisciplinary Recommendations From the Society of Radiologists in Ultrasound Consensus Conference. *Circulation.* 2018;137(14):1505-1515.
Fischer EA, Kinnear B, Sall D, et al. Hospitalist-Operated Compression Ultrasonography: a Point-of-Care Ultrasound Study (HOCUS-POCUS). *J Gen Intern Med.* 2019;34(10):2062-2067.
Pedraza García J, Valle Alonso J, Ceballos García P, Rico Rodríguez F, Aguayo López MÁ, Muñoz-Villanueva MDC. Comparison of the Accuracy of Emergency Department-Performed Point-of-Care-Ultrasound (POCUS) in the Diagnosis of Lower-Extremity Deep Vein Thrombosis. J Emerg Med. 2018 May;54(5):656-664. doi: 10.1016/j.jemermed.2017.12.020. Epub 2018 Jan 3. PMID: 29306580.
Kory PD, Pellecchia CM, Shiloh AL, Mayo PH, DiBello C, Koenig S. Accuracy of ultrasonography performed by critical care physicians for the diagnosis of DVT. Chest. 2011 Mar;139(3):538-542. doi: 10.1378/chest.10-1479. Epub 2010 Oct 28. PMID: 21030490.

3-point compression Ultrasound

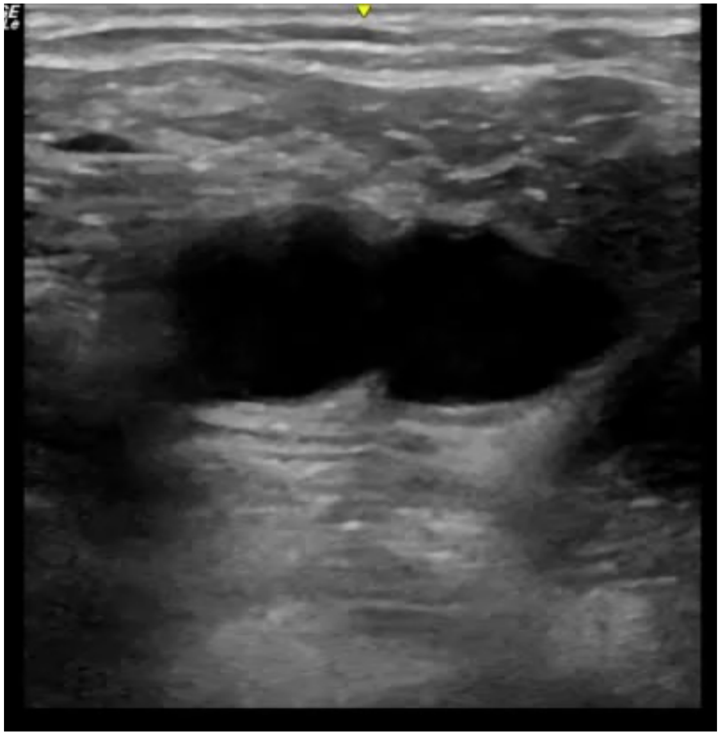

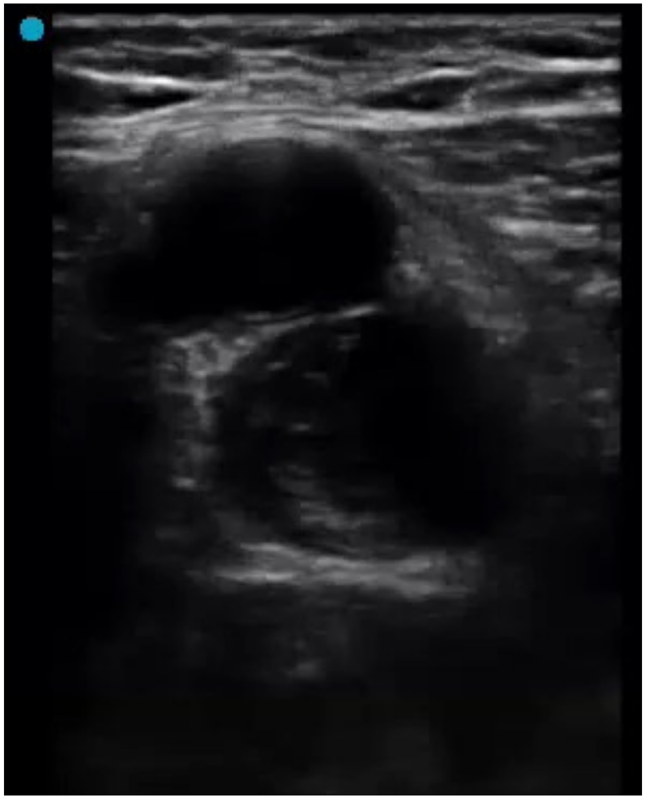

Leg Vasculature Anatomy

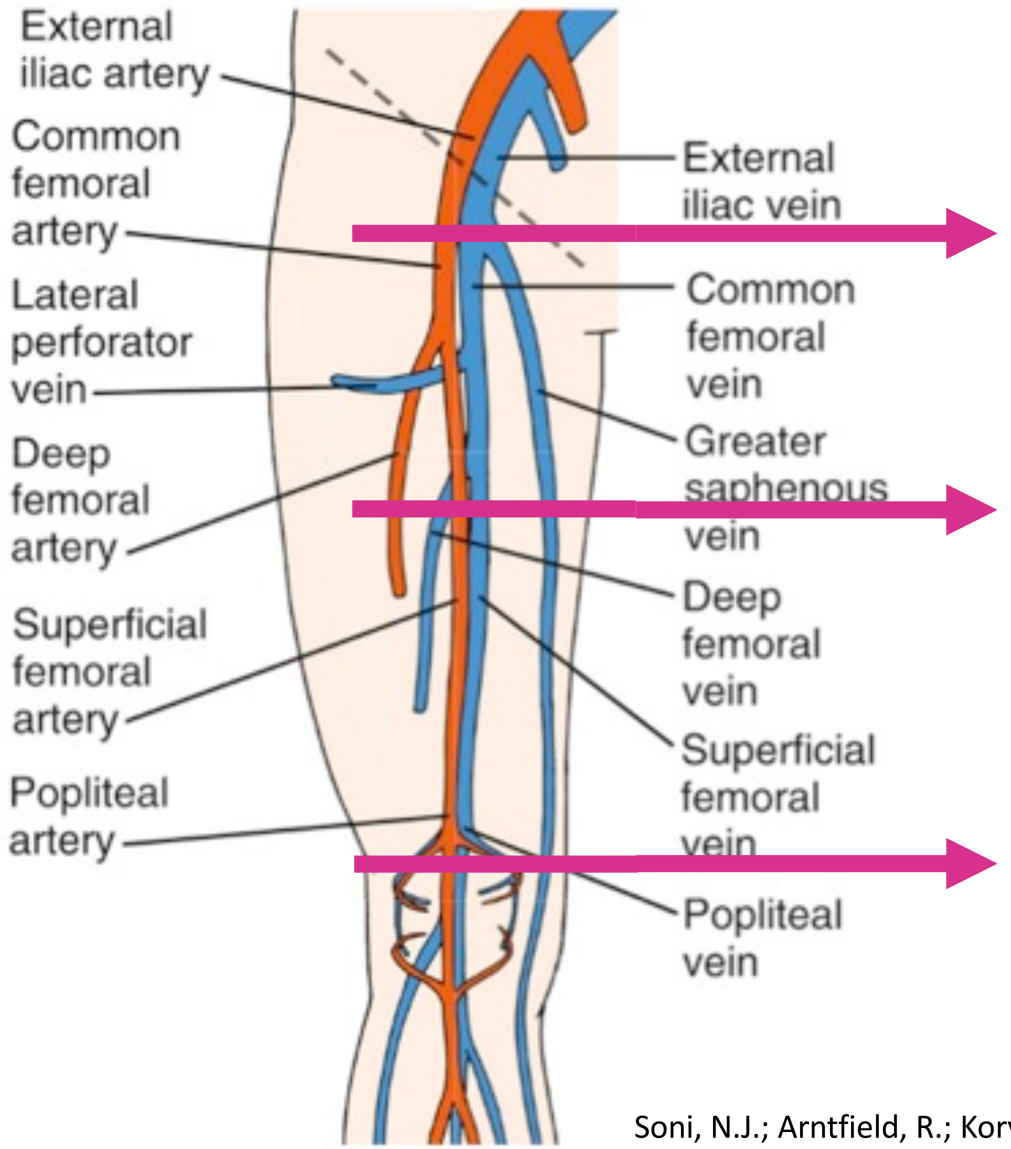

Common Femoral Vein (CFV)

Femoral Vein (FV) aka "superficial femoral vein"

Popliteal Vein (PV)

Soni, N.J.; Arntfield, R.; Kory, P. *Point of Care Ultrasound*, 2nd ed.; Elsevier: Amsterdam, The Netherlands, 2020.

CFV Level

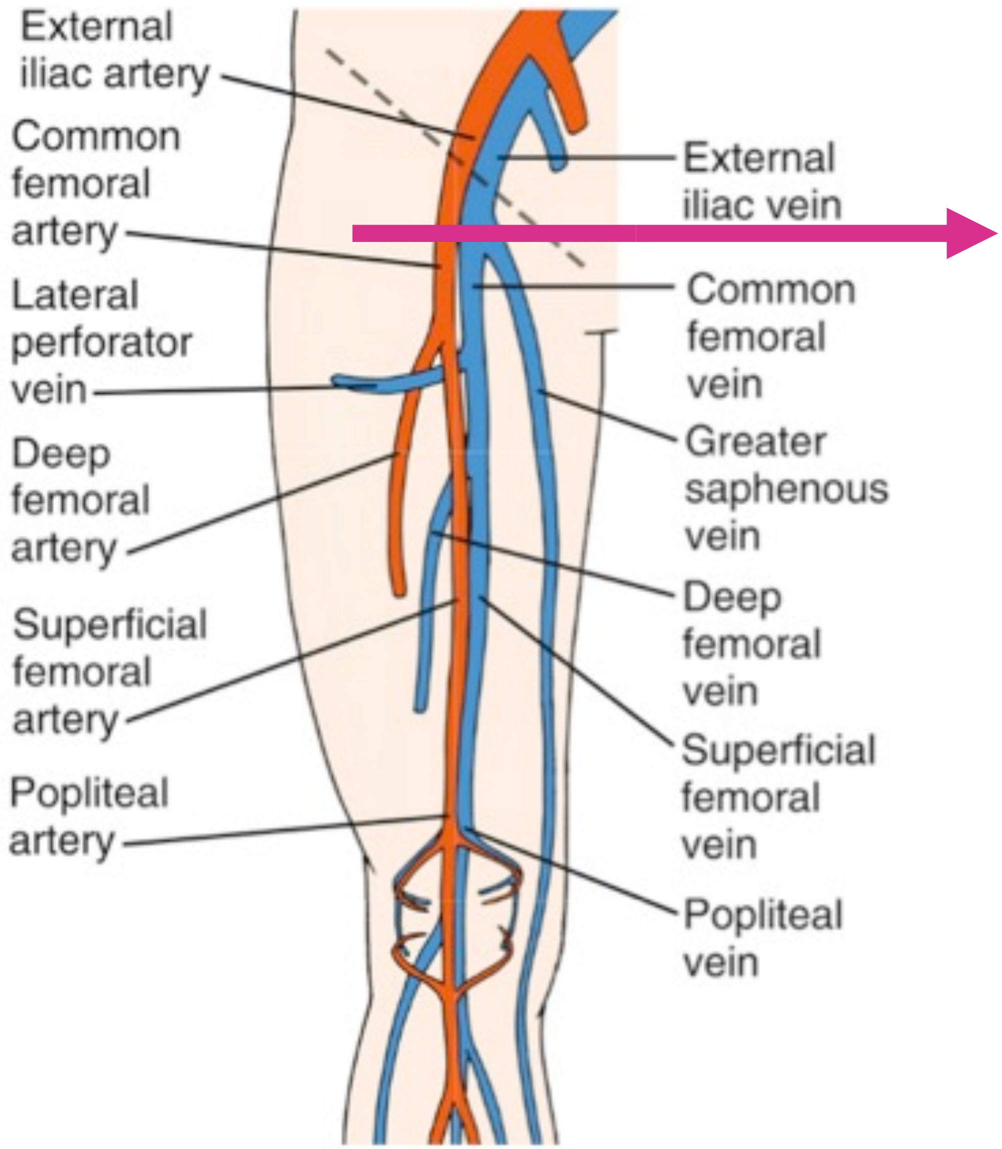

Common Femoral Vein (CFV)

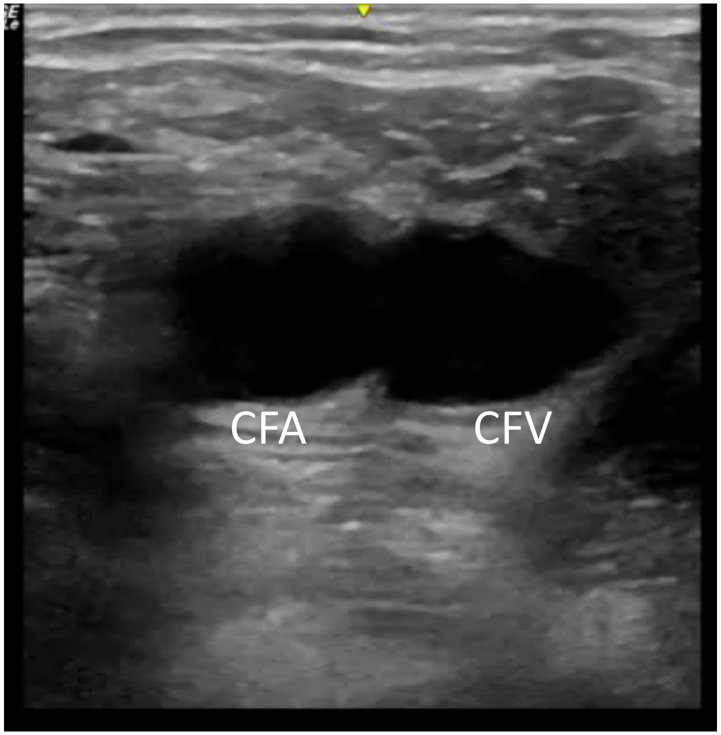

Soni, N.J.; Arntfield, R.; Kory, P. *Point of Care Ultrasound*, 2nd ed.; Elsevier: Amsterdam, The Netherlands, 2020.

CFV Details

- It is easy to capture the branching of the greater saphenous vein on this view!

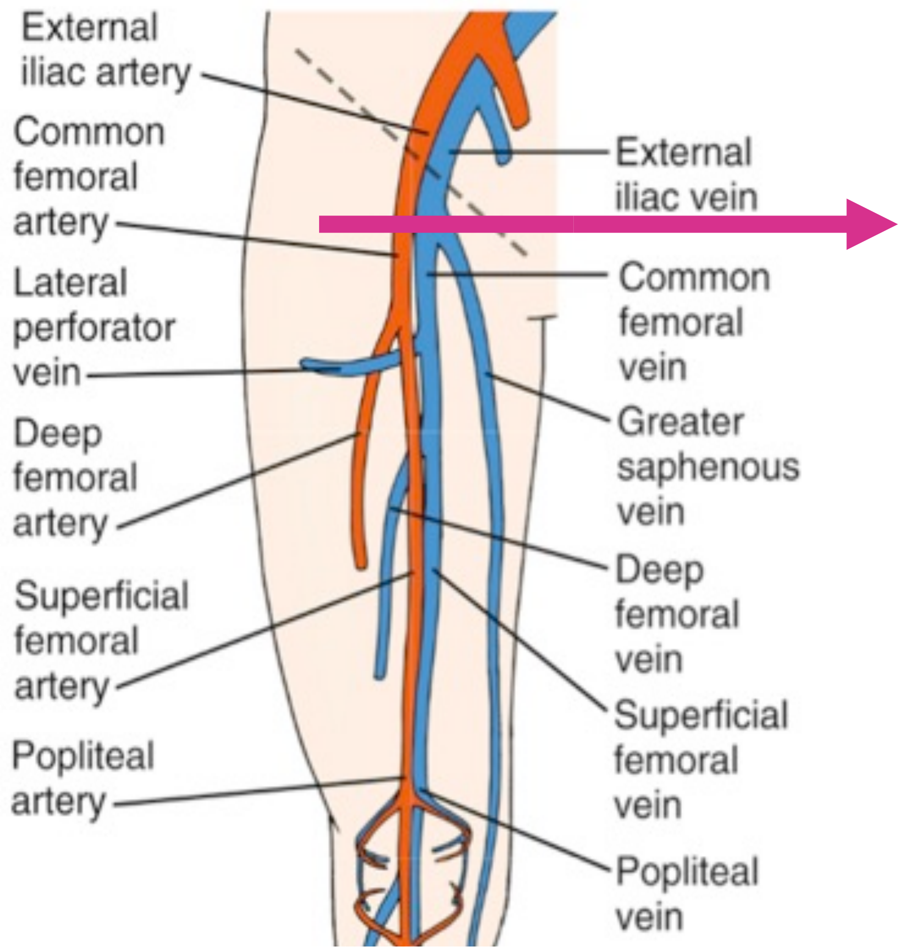

Common Femoral Vein (CFV)

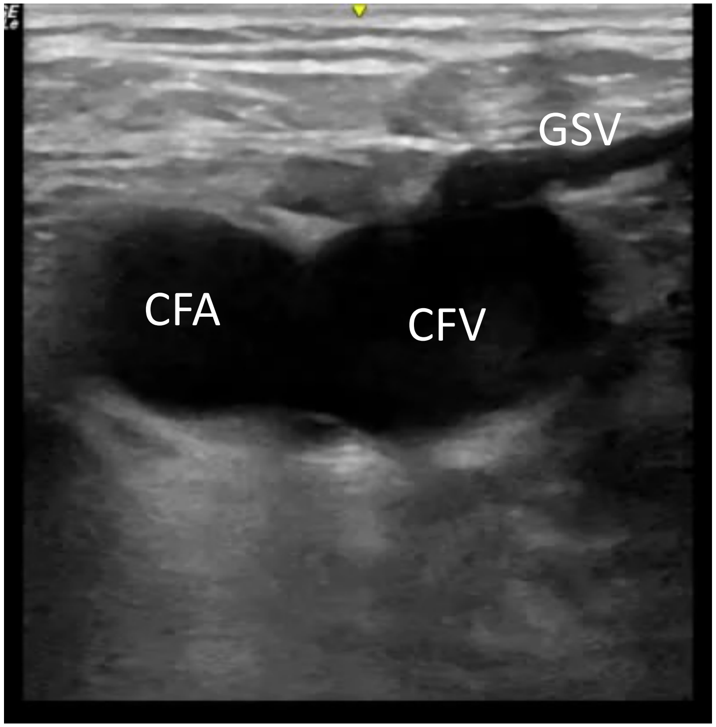

Soni, N.J.; Arntfield, R.; Kory, P. *Point of Care Ultrasound*, 2nd ed.; Elsevier: Amsterdam, The Netherlands, 2020.

FV Level

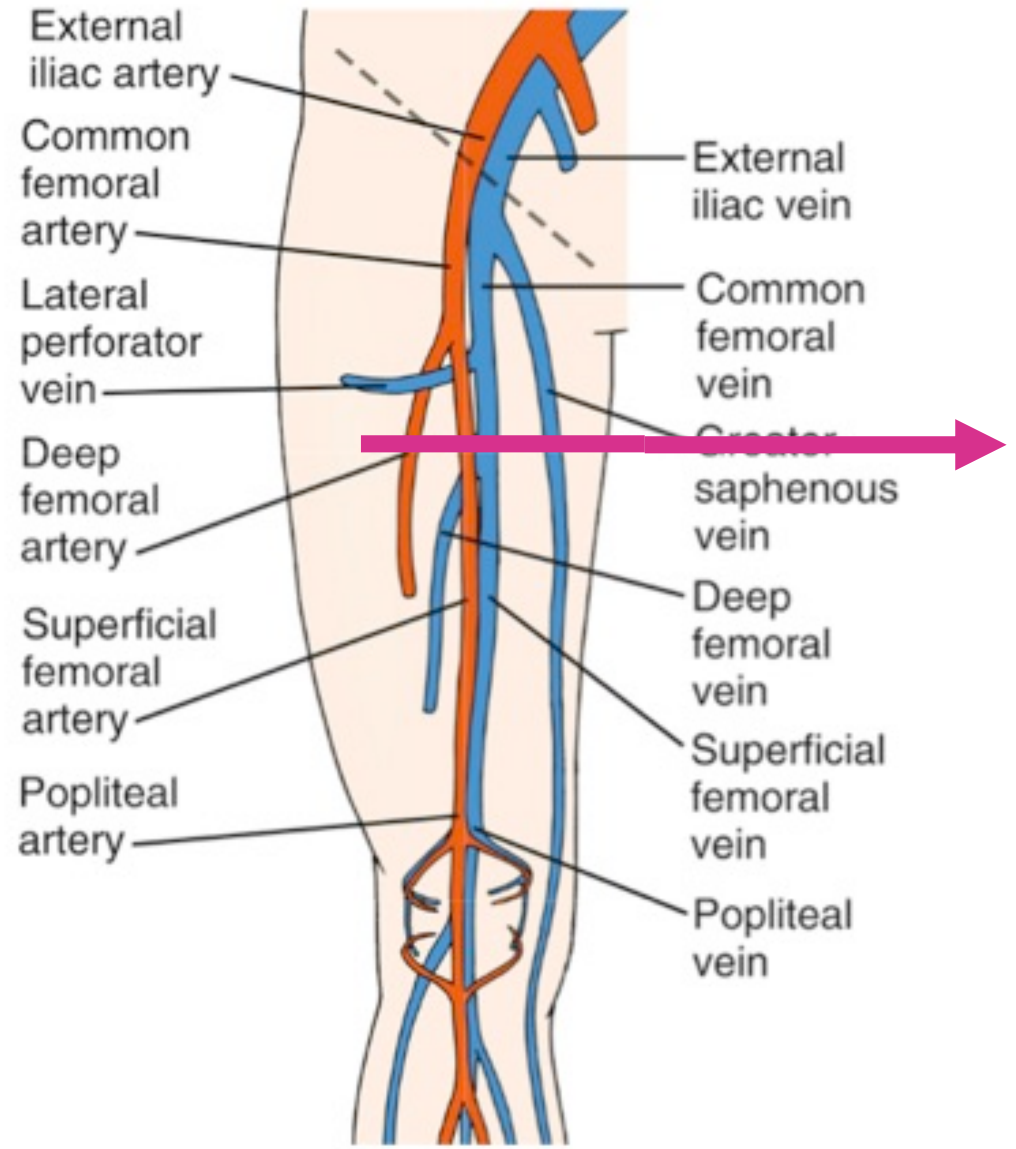

Femoral Vein (FV) aka
“superficial femoral vein”

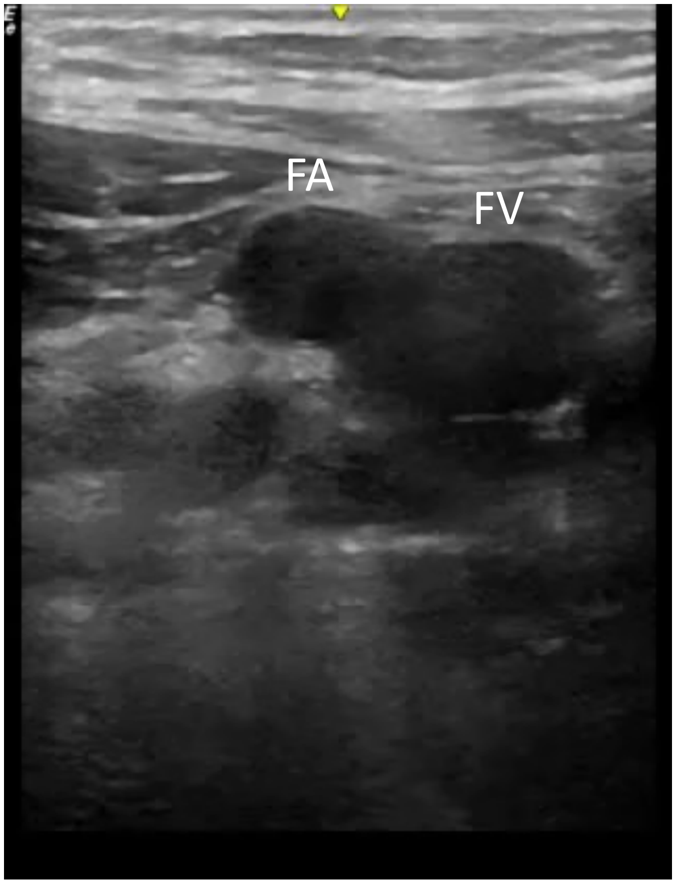

Soni, N.J.; Arntfield, R.; Kory, P. *Point of Care Ultrasound*, 2nd ed.; Elsevier: Amsterdam, The Netherlands, 2020.

FV Details

- This level can be difficult to isolate and is quite proximal to CVF level. The deep femoral vein and artery are also often captured.

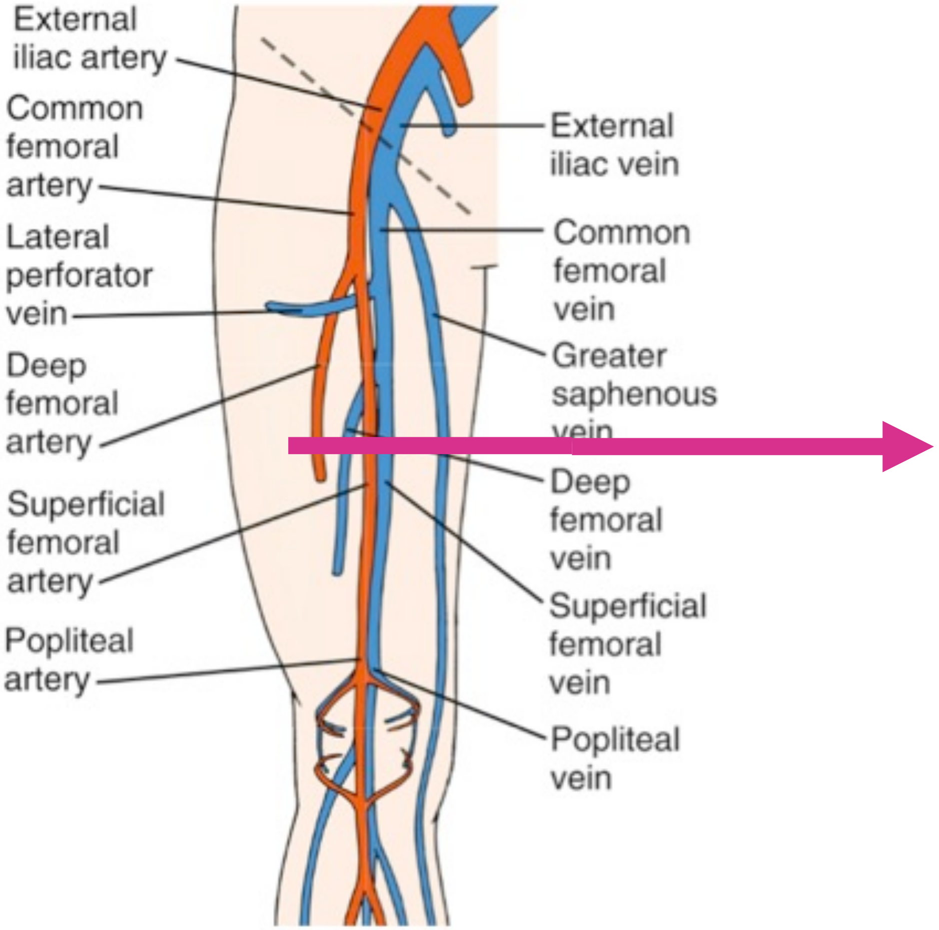

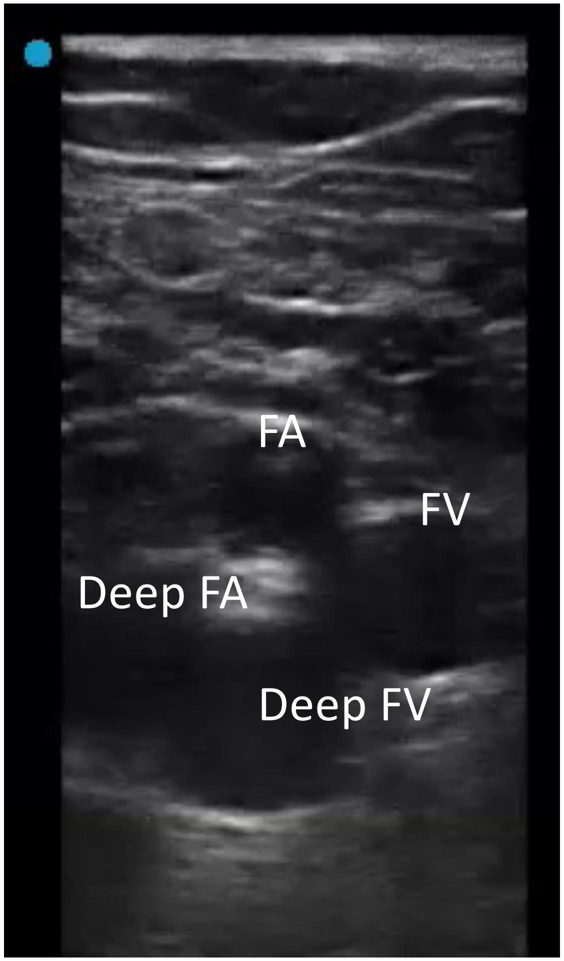

Soni, N.J.; Arntfield, R.; Kory, P. *Point of Care Ultrasound*, 2nd ed.; Elsevier: Amsterdam, The Netherlands, 2020.

FV Level with Positive Scan

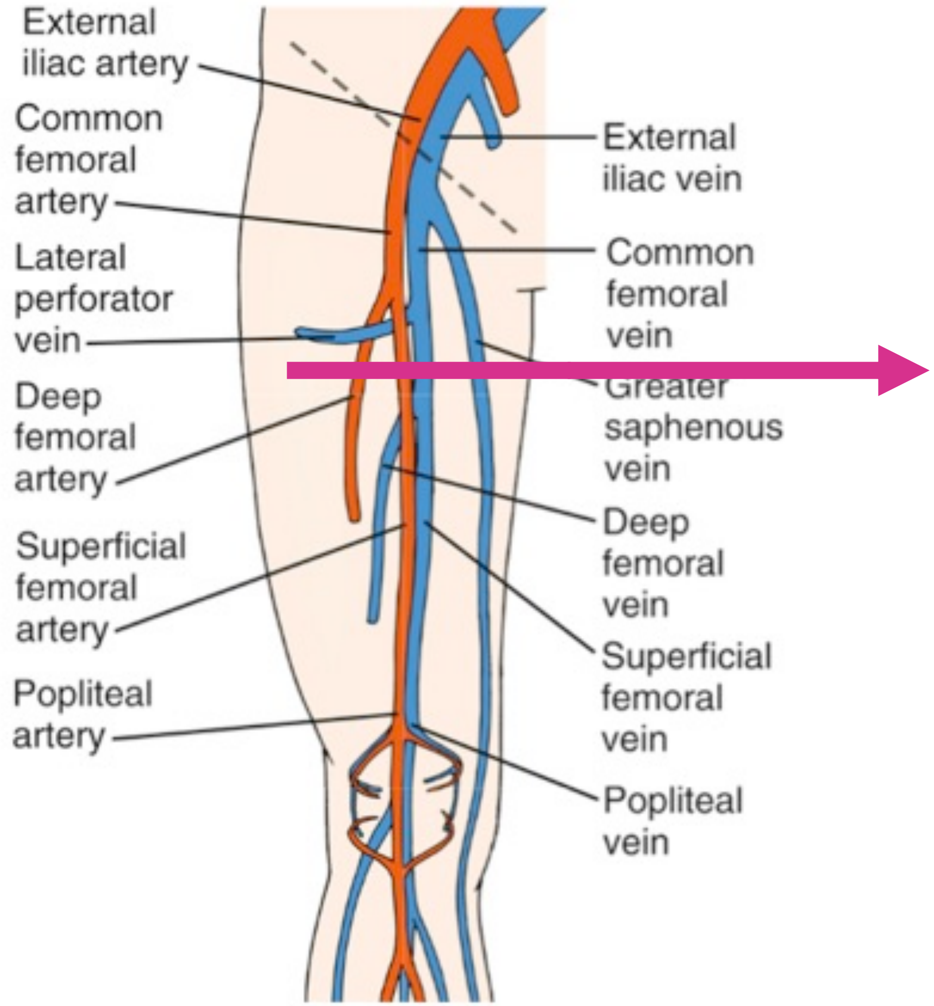

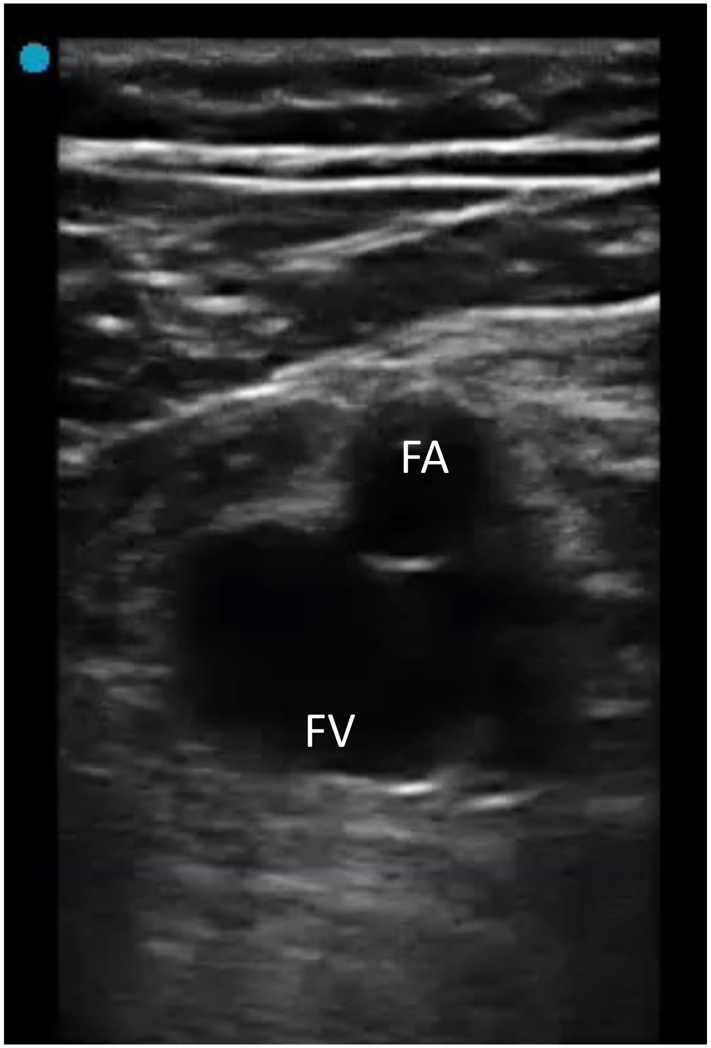

Positive scan on left leg right above junction of femoral vein

PV level

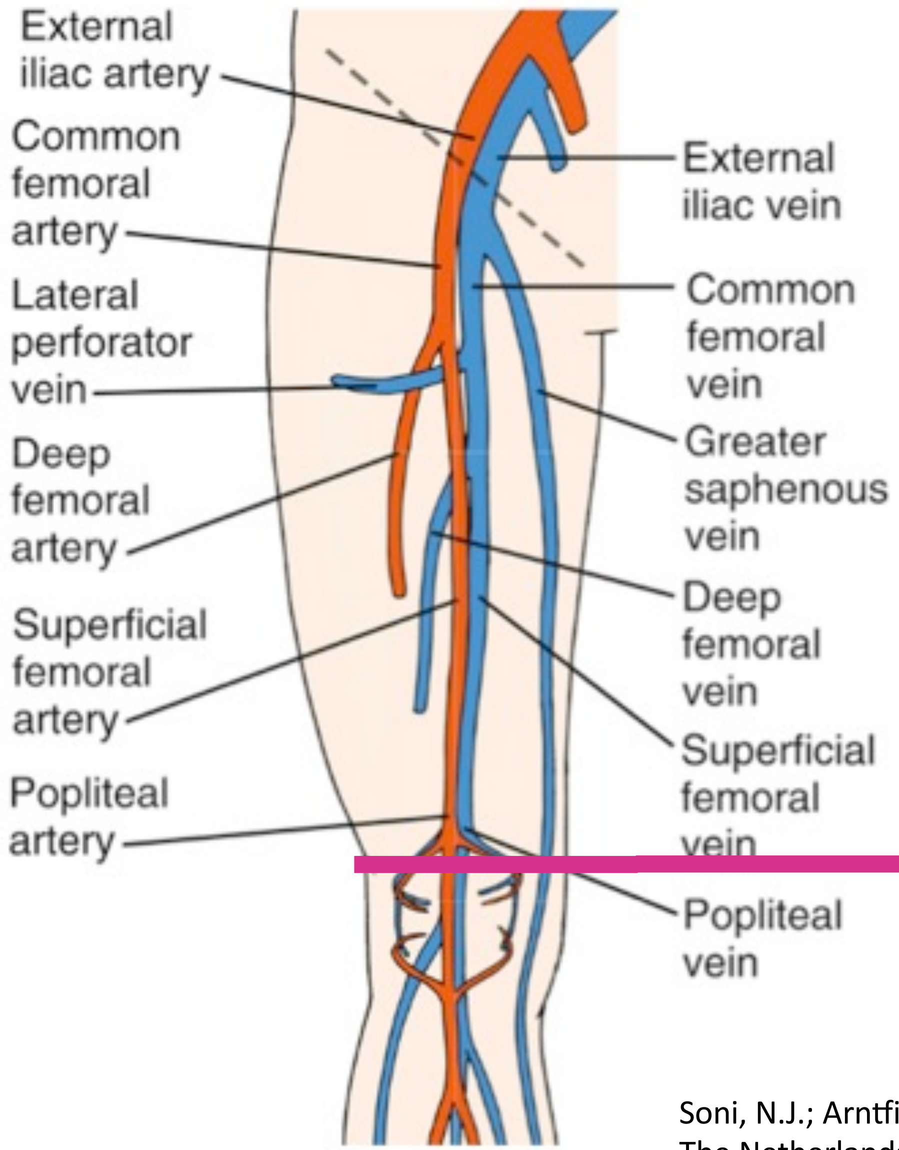

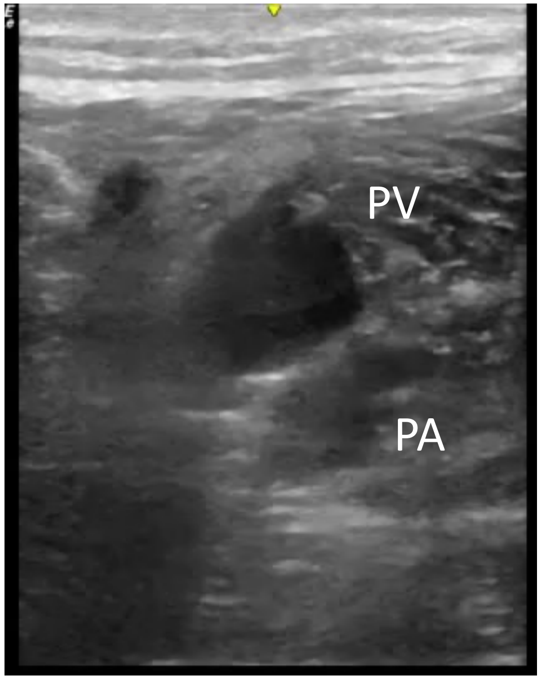

Popliteal Vein (PV)

Soni, N.J.; Arntfield, R.; Kory, P. *Point of Care Ultrasound*, 2nd ed.; Elsevier: Amsterdam, The Netherlands, 2020.

PV level with Positive Scan

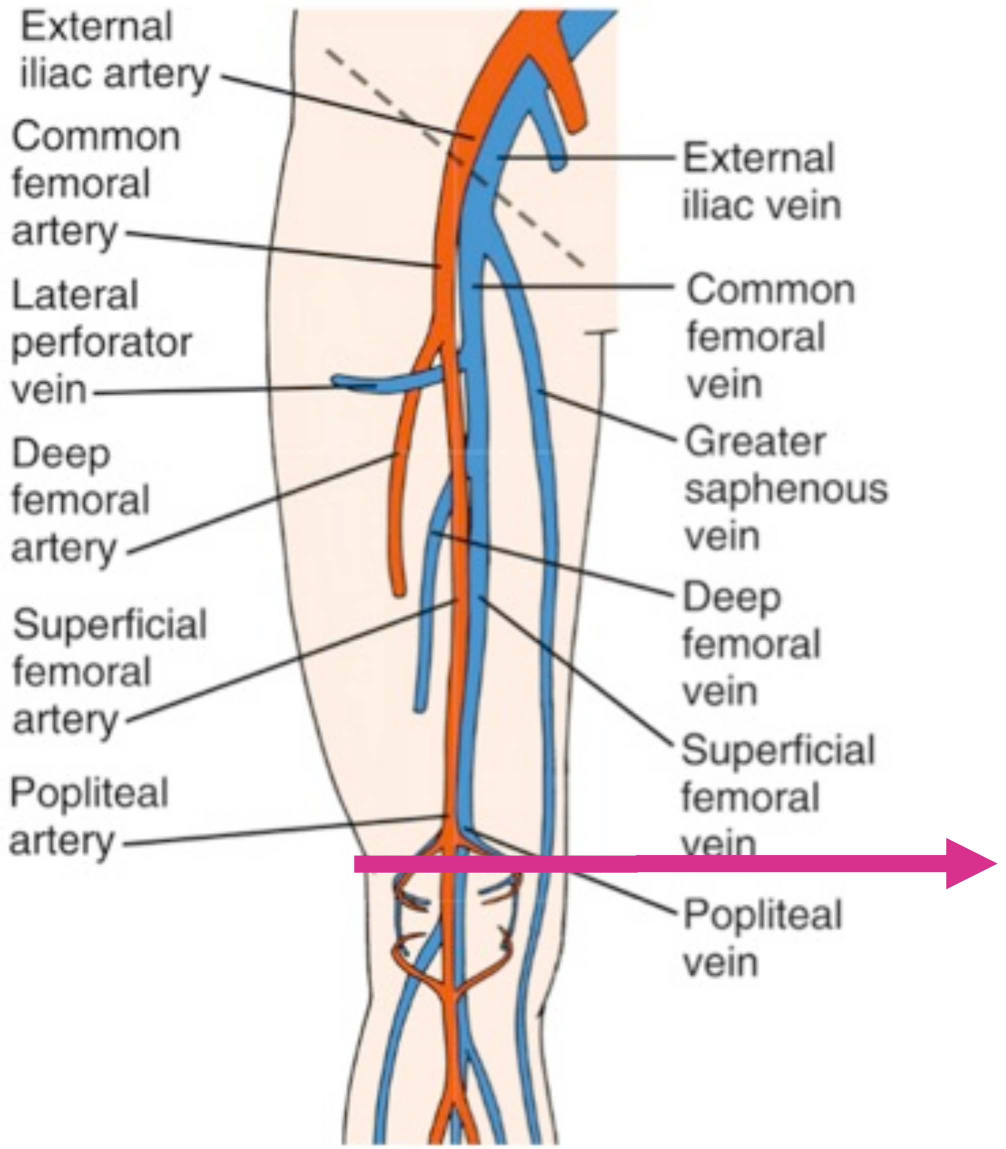

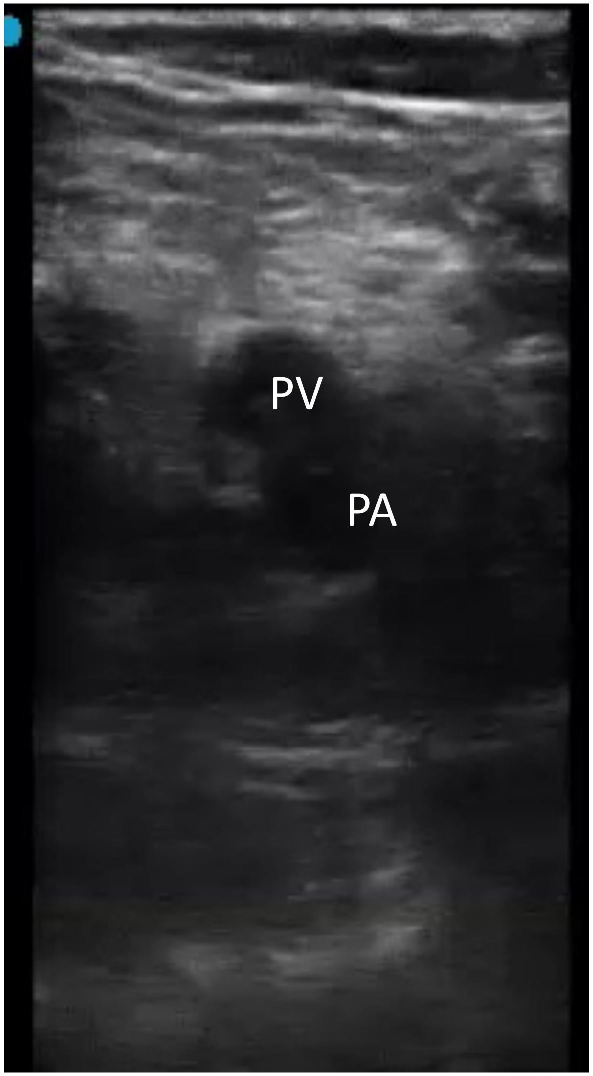

Soni, N.J.; Arntfield, R.; Kory, P. *Point of Care Ultrasound*, 2nd ed.; Elsevier: Amsterdam, The Netherlands, 2020.

3-CUS Protocol

1. Use linear (vascular) probe
2. Adjust ultrasound with indicator to patient's right
3. Place patient in frog-leg position for groin scans and flex leg at 45-degree angle for popliteal fossa
4. Adjust depth to center femoral vessels on screen and adjust gain so that vascular structures are anechoic
5. Identify common femoral vein, femoral vein ("superficial") and popliteal vein at popliteal fossae
6. Compress at all junction points

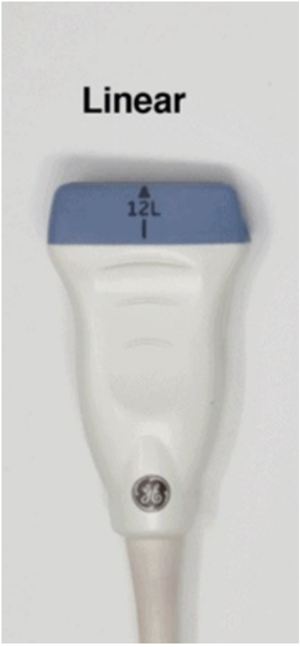

Compressing at > 3 points will only improve accuracy

https://www.pocus101.com/ultrasound-machine-basics-knobology-probes-and-modes/
