# Supplementary material for: An Introductory Curriculum for Internal Medicine Interns in Point-of-Care Ultrasound to Detect Lower Extremity Deep Vein Thrombosis
Source: POCUS J. 2022 Nov 21;7(2):185–6. doi: 10.24908/pocus.v7i2.15937 (PMC9983714; doi:10.24908/pocus.v7i2.15937)
Supplement: Supplementary File S2 [file pocusj-07-15937-s002.pdf]

ID Number: ______
Observed by: _______

Please assess each operator for all the following steps. Operators should verbalize vasculature on at least one leg.

Operators should perform at least 3 points of compression at the common femoral vein, mid/distal femoral vein (superficial) past the junction of the femoral vein and popliteal vein.

1. Uses Vascular (linear) Probe YES ______ NO ______
2. Adjusts ultrasound with screen indicator to left YES ______ NO ______
3. Probe indicator placed to patient's right YES ______ NO ______
4. Positions patient appropriately in frog-leg position for groin scans and flexes leg at a 45 degree angle to scan popliteal fossa. YES ______ NO ______
5. Adjusts depth to center femoral vessels on screen YES ______ NO ______
6. Adjusts gain so that vascular structures are anechoic and appropriate texture is seen in adjacent muscle YES ______ NO ______
7. Identifies common femoral vein (CFV) YES ______ NO _____
8. Identifies greater saphenous vein (GSV) YES ______ NO ______
9. Identifies femoral vein (aka "superficial") YES ______ NO ______
10. Identifies deep femoral vein YES ______ NO ______
11. Identifies popliteal vein YES ______ NO ______
12. Compresses at all junction points YES ______ NO ______

Checklist adapted from J Grad Med Educ. 7(4):567-73 (2015). Step #12 from original checklist ("attempts to compress along entire SFV") removed for simplicity. Junction points which require compression testing are defined as common femoral vein (CFV), superficial femoral vein (SFV) and popliteal vein (PV). Added step #4 given that residents may not be familiar with mechanics of scanning.
